# Brain-on-a-Chip: Dream or Reality?

**DOI:** 10.3389/fnins.2022.837623

**Published:** 2022-03-02

**Authors:** Martina Brofiga, Paolo Massobrio

**Affiliations:** ^1^Department of Informatics, Bioengineering, Robotics, Systems Engineering (DIBRIS), University of Genova, Genova, Italy; ^2^ScreenNeuroPharm, Sanremo, Italy; ^3^National Institute for Nuclear Physics (INFN), Genova, Italy

**Keywords:** brain-on-a-chip, neurotechnologies, network dynamics, neuropharmacology, dissociated cultures, assembloids, neurospheroids, hiPSC

## Introduction

One of the dreams of neuroengineers is building a human brain. More realistically, building an accurate model of the brain with thousands of neurons synaptically connected to be used for a large variety of experiments where populations activity plays a fundamental role. If in the pioneering era of the neurosciences, it was sufficient performing experiments with isolated neurons to understand the single-channel kinetics and the genesis of the action potential, or with couples of neurons to understand synaptic mechanisms, phenomena like cognition and behavior, and the onset of neurological diseases need realistic and reliable models of the human brain. “*To really understand how our own brain works and ‘what makes us human', it is essential to study the human brain at the fine-grain level of cells and the connections that they make with other cells, the synapses*,” argued Idan Segev in 2018 (Eyal et al., 2018). If the computational approach by means of the development of complex mathematical models is a possibility (Markram, 2006), the recent advances in stem cell knowledge and in neurotechnologies paved the way to (partially) recreate the human brain *in vitro* and to record its electrophysiological activity. After more than 10 years from its first appearance, the term *brain-on-a-chip* can now be appropriately used (Wheeler, 2008). Nowadays, we are able to replicate many human neuronal types and peculiar brain regions in the form of engineered neuronal cultures, like neurospheroids or brain organoids, directly from embryonic and human induced pluripotent stem cells (hiPSC), and to couple them to a technological counterpart (i.e., chip).

Thus, how far can we go with “new generation” brain-on-a-chip models to unravel the many hidden mechanisms of the brain? Which experiments will benefit from this model? Are we able to “clone” our brain (or a brain region) to use it as a backup in case of severe impairments? Starting from the recent advances in the field of neuroengineering and neurotechnologies, we will discuss what should be done, which questions can be answered by using brain-on-a-chip models, and what will continue being a dream and what could become reality in the next decades.

## Which Applications For Brain-on-a-Chip?

Advances in medicine and the spread of health facilities have lengthened life expectancy and brought to a drop in birth rates with a consequent mass aging of the population. In 2020, more than 147 million people around the world (equal to 1.9% of the world population) were between 80 and 99 years old (infographic of Visual Capitalist). Aging is accompanied by an increase in the burden of non-communicable diseases, including neurodegenerative ones resulting from the gradual and progressive loss of neuronal cells that leads to the onset of nervous system dysfunctions. According to the National Institute of Neurological Disorders and Stroke, there are more than 600 known neurological disorders, with about 50 million Americans affected each year (Brown et al., 2005). Such diseases cost the US economy billions of dollars each year: about 100 billion dollars are spent on Alzheimer's disease alone (Meek et al., 1998). In 2010, it was estimated a cost of about 800 billion euros for managing brain disorders in Europe (Olesen et al., 2012). Furthermore, the development of drugs for the treatment of brain diseases is more difficult than the development of drugs in other therapeutic areas. “*Central Nervous System (CNS) drugs face greater development challenges compared to non-CNS drugs due, in large part, to a poor understanding of the underlying pathophysiology of many of the disorders they seek to treat, as well as difficulty identifying and measuring appropriate clinical endpoints*,” wrote Joseph A. DiMasi, director of economic analysis at Tufts Center for the Study of Drug Development. Such limits define longer time frames and higher costs during the development and approval process of CNS drugs, making the field less attractive to investors. Several pharmaceutical companies have downsized their research divisions in neuroscience (Choi et al., 2014): GlaxoSmithKline in 2010 abandoned research in this field; after Merck also Pfizer in 2018 stopped research for Alzheimer's and Parkinson's diseases medicines. The high costs are often linked to drug failure at an advanced stage of the experimental process. Thus, if on the one hand, there is a tremendous need of methods to treat neurological disorders, on the other, it is needed to reduce the costs. In this scenario, the development of realistic *in vitro* models of the brain plays a fundamental role not only in basic research but also in more fruitful applications spanning from drug discovery up to impaired brain models.

Until now, the *in vitro* models used in pre-clinical tests are oversimplified, considering two-dimensional, homogeneous (i.e., only one cell type), and often of animal origin (e.g., murine neurons) cultures, characteristics that make these models far from the human brain. Such oversimplification often under- or over-estimates the response to the drug, leading to biased information (Bang et al., 2019). The human brain is made up of about 86 billion neurons that differ in structure and function (heterogeneity) organized according to precise connectivity rules (modularity) in a three-dimensional (3D) fashion. These three keywords set the concept of brain-on-a-chip: an engineered system in which neurons can live, grow, and connect to establish intricate connectivity coupled to integrated micro-transducers. This hybrid structure should ensure the recording of electrophysiological activity and the monitoring of other relevant parameters like neurotransmitters concentrations, metabolic alterations, as well as allowing optical investigations with calcium-imaging or immunofluorescence techniques (Weisenburger and Vaziri, 2018). The different approaches followed during the years (for a review see (Brofiga et al., 2021b) and references therein) allowed the definition of heterogeneous models and the analysis of the interaction between different brain regions. Examples are the cortico-striatal (Virlogeux et al., 2018), the cortico-hippocampal (Brofiga et al., 2021c), the cortico-thalamic (Brofiga et al., 2021a), and the cortico-amygdala-hippocampal (Dauth et al., 2017) circuits. However, in these 2D models, the rigid substrate does not mimic the deformability of the extracellular matrix (ECM) observed *in vivo*. Neurons perceive the rigidity of the surrounding environment and modify their gene expression profile in response (Baker and Chen, 2012). Furthermore, 2D cultures lack endogenous 3D cell-cell interactions and physiological signals provided *in vivo* by the ECM. In the *in vitro* development process of brain-on-a-chip models, a fundamental step is represented by the engineering and the tailoring of the 3D matrix, containing chemical and mechanical signals suitable for mimicking the native ECM. Typically, the scaffolds make use of natural biomaterials due to their biocompatible and biodegradable properties [for a review, see (Antill-O'Brien et al., 2019) and references therein].

Over the years, neuroscientists have used animal models to test the safety of new drugs to treat brain disorders. Although the historical value of animal experimentation is indisputable, some pressure groups question the legitimacy of its use for the advancement in biomedical knowledge and the development of new diagnostic and therapeutic treatments. Mice/rats have evolutionarily conserved their brains, i.e., they have very similar architectures made up of similar types of brain cells as humans. However, there is often an evident opposition of behavior in the development of a new drug, since it can have the desired effects on mice but can fail when humans are treated. A study conducted at the Allen Institute for Brain Science in Seattle found a possible answer. Brain cells in mice activate very different genes from human brain cells (Peng et al., 2021): serotonin is a neurotransmitter that regulates appetite, mood, memory, and sleep by binding to specific brain cells. However, serotonin receptors are not found on the same cells in murine. Thus, a drug that increases serotonin levels, such as those used for depression, could act on very different cells in humans and mice. This diversity could affect not only pre-clinical tests on animals but also preclinical tests *in vitro*, hence the need to use a different approach.

## The Next-Generation Brain-on-a-Chip

**Figure 1:**
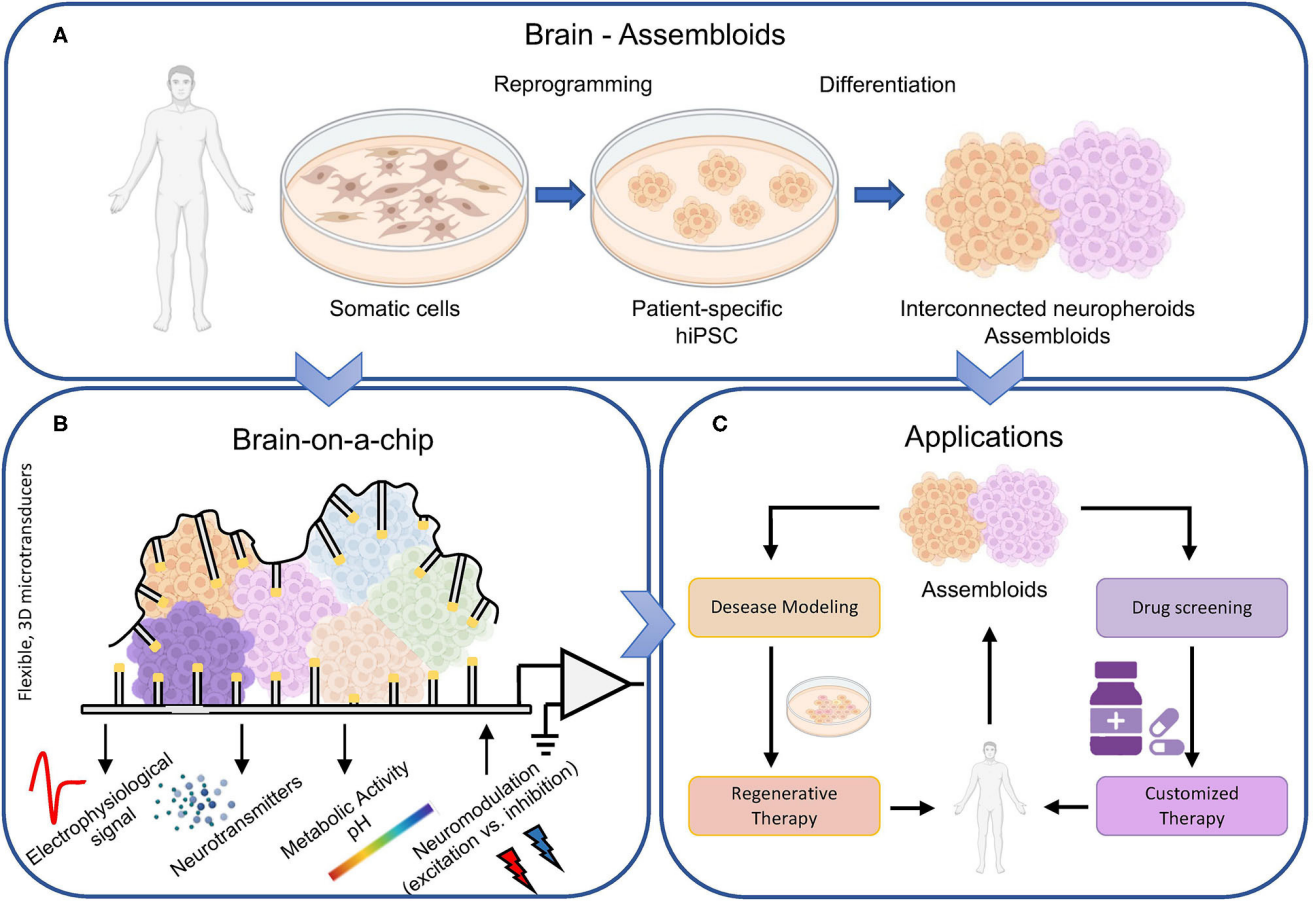
Brain-on-a-chip overview. **(A)** The biological component of next generation brain-on-a-chip models come directly from the individuals. The reprogramming of human somatic cells in hiPSC allows the definition of different neuronal types, maintaining the fundamental topological properties of the brain, i.e., the 3D structure, a sufficient level of heterogeneity, and a modular connectivity. Thus, interconnected neurospheroids and assembloids can be cultivated *in vitro* and **(B)** coupled to microtransducers able not only to record the electrophysiological activity of the biological structure, but also to monitor other relevant parameters like neurotransmitters concentrations and variations of the metabolic activity. In addition, such devices should be bi-directional, i.e., able to modulate the electrophysiological activity by delivering excitatory or inhibitory stimuli. **(C)** Such accurate biological model of the brain can be exploited not only for basic science but also for drug screening (to obtain customized and patient-specific therapy), as well as to study *in vitro* the pathogenesis of brain disorders and consequently try finding solutions.

The introduction of pluripotent stem cells has opened new horizons for *in vitro* engineered models for understanding the genesis and the development of neurodegenerative diseases. In 2006, it was demonstrated the possibility to reprogram mature human somatic cells into induced pluripotent stem cells (hiPSC) (Takahashi and Yamanaka, 2006). HiPSCs retain the genetic characteristics of their donors, which allow genotype-dependent pathophysiology to manifest at cellular level to recapitulate pathological biomarkers, and thus support studies on genetic disorders (Tang et al., 2015). Without any doubt, such a finding paved the way to cultivate *in vitro* the patient cells (Figure 1A). Their use has made possible to investigate the physiological and pathological behavior of tissues and cell types, like the ones of the nervous system, that are not accessible in a non-invasive way. The choral synergy of disciplines like tissue engineering, biochemistry, and neuroscience allowed recreating the right *in vivo* microenvironment, where cells can grow in a 3D topology thanks to a self-assembling approach. Such 3D neuronal cultures, also known as organoids or organ spheres, maintain many attributes of human *in vitro* development. Neurospheres (spheroids made up of induced neuronal cells) are able to reproduce functions of the developing brain, such as proliferation, migration, and differentiation (da Silva Siqueira et al., 2021). It is worth noticing that during development, the formation of specific brain regions is shaped by interactions with other brain regions. In 2017, Birey and coworkers obtained a hiPSC-derived 3D model of the dorsal and ventral forebrain (Birey et al., 2017). The authors assembled subdomain-specific forebrain spheroids *in vitro* and recreated a micro-physiological system containing both functionally integrated glutamatergic and GABAergic neurons. In addition, the use of such assembloids allowed identifying transcriptional changes associated with interneuron migration and modeling otherwise inaccessible pathological processes. In particular, the same authors found an overexpressed migration of GABAergic neurons in the case of the Timothy syndrome, a very rare form of autism (Hesse et al., 2008). In this perspective, the use of assembloids is crucial to model complex cell-cell interactions, for the study of specific neuronal circuits (Andersen et al., 2020), and to evaluate easier than in the *in vivo* brain possible mutations induced by impairments. Scaling up toward more complex forms of 3D structures, brain organoids are other examples that allowed the definition of interconnected brain regions like the midbrain with the striatum and the cortex with the striatum (Miura and Pasca, 2021).

However, considering only 3D models of interacting neurons it is not enough. In order to investigate the genesis as well as the progression of neurodegenerative diseases and to appreciate the effects of new drugs, the blood-brain barrier (BBB) should also be embedded in a brain-on-a-chip model. Although the BBB allows the treatment of peripheral pathologies with drugs without them affecting the CNS, it makes it difficult to ascertain the efficacy of drugs for therapeutic use in diseases of the CNS. Furthermore, the weakening of the BBB could favor the onset of infections in nervous tissues, which represent the fulcrum of many neurodegenerative diseases (Sweeney et al., 2019).

The progresses in the biological domain require similar advancements in the micro/nanotechnologies field to keep up with the more complex biological models (Figure 1B). If 10 years ago, devices with thousand of microelectrodes (Berdondini et al., 2009; Frey et al., 2009) constituted a breakthrough in the investigation of the computational properties of large-scale planar neuronal cultures, now it is time for a further step forward. Next generation brain-on-a-chip should implement technologies to allow the monitoring of physiological properties (not only the electrophysiology) of 3D biosystems (neurospheroids, assembloids). Conventional technologies with rigid substrate and planar electrodes provide functional interfaces only to small areas of the 3D culture and do not allow untangling what happens in the hidden layers of the structure itself. In this perspective, Park and colleagues presented a novel device composed by a soft and shape-matched semiconductor platform that can wrap the 3D cultures. This device supports multifunctional electronic, optoelectronic, thermal, mechanical, and biochemical interfaces, ensuring the extraction of electrophysiological information with greater detail and efficiency (Park et al., 2021). Similarly, in 2021, Shin and coworkers developed a device capable of simultaneously recording the electrophysiological activity of 3D *in vitro* models using 63 electrodes distributed over 18 vertical shanks. The device allows stimulating the culture both chemically and optically in a localized and selective way (Shin et al., 2021).

## Perspective Toward Precision Medicine

Are we able to replicate a brain *in vitro*? In 2022, the answer is definitively no. Today and for the next (at least) 10 years, it would be false and misleading to promise an exact replica of a brain. More honestly, we can assert that the current state of the art allows recreating some brain areas that can be monitored with innovative devices. The key for next generation brain-on-a-chip is the coexistence of innovative micro/nanotechnologies (artificial component) and 3D biosystems (biological counterpart) obtained with novel neuroengineering techniques. The artificial component should include multi-functional recording devices for the monitoring not only of the electrophysiological activity, but also of relevant parameters related to metabolism and biochemicals reactions. Nonetheless such devices should embed the possibility to actively interact with the biological model by modulating its electrophysiological activity with excitatory or inhibitory stimuli (neuromodulation). Alternatively to the conventional electrical stimulation, the neuromodulation by optogenetics has the great advantage for cell-specificity, high spatial resolution, and non-invasiveness, but it requires the genetic modifications of neurons by means of viruses (Spangler and Bruchas, 2017). In the future, neuromodulation techniques could exploit the recent advancements of photothermal neural stimulation methods (Lee et al., 2018). Photothermal stimulation makes use of mid- or far-infrared light and (potentially) metal nanoparticles, which generate heat by absorbing a specific light wavelength through local surface plasmonic resonance. This approach has the great advantage to either excite or inhibit neuronal activity in specific illuminated areas (Yoo et al., 2014, 2016) with a higher spatial and temporal resolution than the conventional electrical one. On the other hand, the 3D biosystems should employ human-cell derived neurons assembled to form interconnected heterogeneous populations. The use of personalized hiPSC in such models will facilitate the discovery of patient-specific treatment strategies as well as unraveling the cell-type specific contribution to a brain disease. In summary, addressing the priorities of patient-specific applications (i.e., precision medicine) requires moving beyond animal models by proposing engineered *in vitro* systems with greater similarity to *in vivo* human brain tissue. The field is truly moving toward precision medicine, but only recently 3D neuronal networks have been used as platforms for investigating brain (dys)functions and complementing *in vivo* animal studies as reviewed in (Amin and Paşca, 2018) and references therein. The possibility to create a personalized ‘*in vitro brain model*' will allow the screening for novel drugs and pre-clinical testing of novel compounds in patients that are resistant to existing medication (Miccoli et al., 2019) (Figure 1C). In conclusion, next generation brain-on-a-chip will favor and enhance the clinical translation.

## Author Contributions

MB and PM conceptualized and wrote the manuscript. All authors contributed to the article and approved the submitted version.

## Conflict of Interest

MB was employed by ScreenNeuroPharm. The remaining author declares that the research was conducted in the absence of any commercial or financial relationships that could be construed as a potential conflict of interest.

## Publisher's Note

All claims expressed in this article are solely those of the authors and do not necessarily represent those of their affiliated organizations, or those of the publisher, the editors and the reviewers. Any product that may be evaluated in this article, or claim that may be made by its manufacturer, is not guaranteed or endorsed by the publisher.
